# Serine Protease Inhibitor Kazal Type 1 (SPINK1) c.194+2T > C Mutation May Predict Long-term Outcome of Endoscopic Treatments in Idiopathic Chronic Pancreatitis

**DOI:** 10.1097/MD.0000000000002046

**Published:** 2015-10-30

**Authors:** Chang Sun, Mu-Yun Liu, Xiao-Gang Liu, Liang-Hao Hu, Tian Xia, Zhuan Liao, Zhao-Shen Li

**Affiliations:** From the Department of Gastroenterology, Changhai Hospital, Second Military Medical University (CS, M-YL, L-HH, TX, ZL, Z-SL); and Changhai Hospital, Second Military Medical University Shanghai, China (X-GL).

## Abstract

Endoscopic interventional is a commonly used treatment method for idiopathic chronic pancreatitis. Serine protease inhibitor Kazal type 1 (SPINK1) 194+2T>C mutation is most frequently observed in Chinese pancreatitis patients and influences the clinical course of idiopathic chronic pancreatitis patients. We conducted this study to determine the impacts of this mutation on the outcome of endoscopic treatments.

In this study, we enrolled 423 patients. Among them, 101 idiopathic chronic pancreatitis patients without other relevant mutations had a successful endoscopic procedure and completed follow-up. Clinical characteristics including Izbicki pain score, exocrine and endocrine function, were evaluated. Genetic sequencing was conducted to detect SPINK1 194+2T>C mutations.

The c.194+2T>C mutation was found in 58 (57.43%) patients. Factors relevant to pain relief are c.194+2T>C mutation (*P* = 0.011), severe pain before treatment (*P* = 0.005), and necessary subsequent endoscopic treatments (*P* < 0.001). More patients with the intronic mutation had deteriorated endocrine function (*P* = 0.001) relative to those patients without the mutation.

Patients carrying the c.194+2T>C mutation were less likely to achieve pain relief through endoscopic treatments. They also have a higher risk of endocrine function deterioration. SPINK1 c.194+2T>C mutation may be applied as a pretreatment predictor in idiopathic chronic pancreatitis patients.

## INTRODUCTION

Chronic pancreatitis (CP) is a progressive inflammatory disease caused by various factors, in which the pancreatic secretory parenchyma is destroyed and replaced by fibrous tissue, eventually leading to the impairment of the exocrine and endocrine functions of the organ.^1^ The main symptom of CP is pain, which is highly variable among individuals presenting with continuous pain of varying severity, and intermittent or background continuous pain coupled with intermittent flares.^2,3^ The specific determinant for this heterogeneity has not been clearly explained yet. All the therapeutic efforts including endoscopic and surgical treatments are mostly aimed at extracting stones and decompressing pancreatic ducts to achieve ideal drainage of the pancreatic duct.^4,5^ Endoscopic retrograde cholangiopancreatography (ERCP) and/or extracorporeal shockwave lithotripsy (ESWL) are often used as a first-step choice in the management main pancreatic duct (MPD) in chronic pancreatitis.^6^ Previously reported data have suggested that 55.0% to 87.5% of patients achieved pain relief during follow-up.^7–9^

The etiologies of chronic pancreatitis include alcoholism, hyperlipidemia, obstructive damage caused by trauma or congenital anomalies, hereditary pancreatitis, autoimmune pancreatitis, and idiopathic pancreatitis according to the classic TIGAR-O system.^10^ Idiopathic chronic pancreatitis (ICP) is more commonly reported in Asian countries as compared to Western countries.^11,12^ Without detectable environmental pathogenic factors, genetic predisposition is considered the predominant etiology of ICP.^13^ Serine protease inhibitor Kazal type 1 (SPINK1) gene (OMIM ∗167790) mutations, which weaken the first-line defense against premature trypsinogen activation, are among the most frequently identified alterations in ICP patients.^14^ The p.N34S mutation has been found worldwide in both CP patients and in healthy controls and is believed to increase the risk of idiopathic pancreatitis 15-fold.^15,16^ Patients with SPINK1 N34S mutations were more likely to develop a dilated duct, calcifications, and diabetes over time than patients with human cationic trypsinogen (PRSS1) mutations.^17^ Recently, the SPINK1 intronic mutation 194+2T>C (IVS3+2T>C) has been found in 19% to 45% of ICP patients in studies conducted in Asian in contrast with 1% to 3% in European studies.^13,18^ We have previously shown that 44.9% in ICP patients and 57.3% in pediatric ICP patients carried the intronic mutation, whereas none of the healthy controls did. Further analysis has suggested the potential relationship between the mutation and early diabetes onset, as well as with a relatively high rate of pancreatic duct stone formation.^19,20^

Considerable evidence has been presented indicating that the SPINK1 194+2T>C (IVS3+2T>C) mutation is an independent impact factor for the prognosis of ICP. However, no studies have focused on the potential predictable role of genetic predisposition during endoscopic treatments for ICP. With the goal of contributing to a better individualized treatment regime, we conducted this study to evaluate whether the SPINK1 194+2T>C mutation independently impacts the outcome of endoscopic treatments for ICP.

## MATERIAL AND METHODS

### Subject Enrollment

All consecutive patients diagnosed with chronic pancreatitis that received treatments between February 2009 and March 2012 at the Department of Gastroenterology at Changhai Hospital of the Second Military Medical University were included in the study. Among them, 423 CP patients agreed to peripheral blood collection for genetic sequencing and subsequent follow-up. After excluding patients who had definitive etiological factors, such as alcohol consumption, heavy smoking, hypercalcemia, pancreatic trauma, and a positive family history, 239 patients got a a presumptive diagnosis of ICP.

**FIGURE 1:**
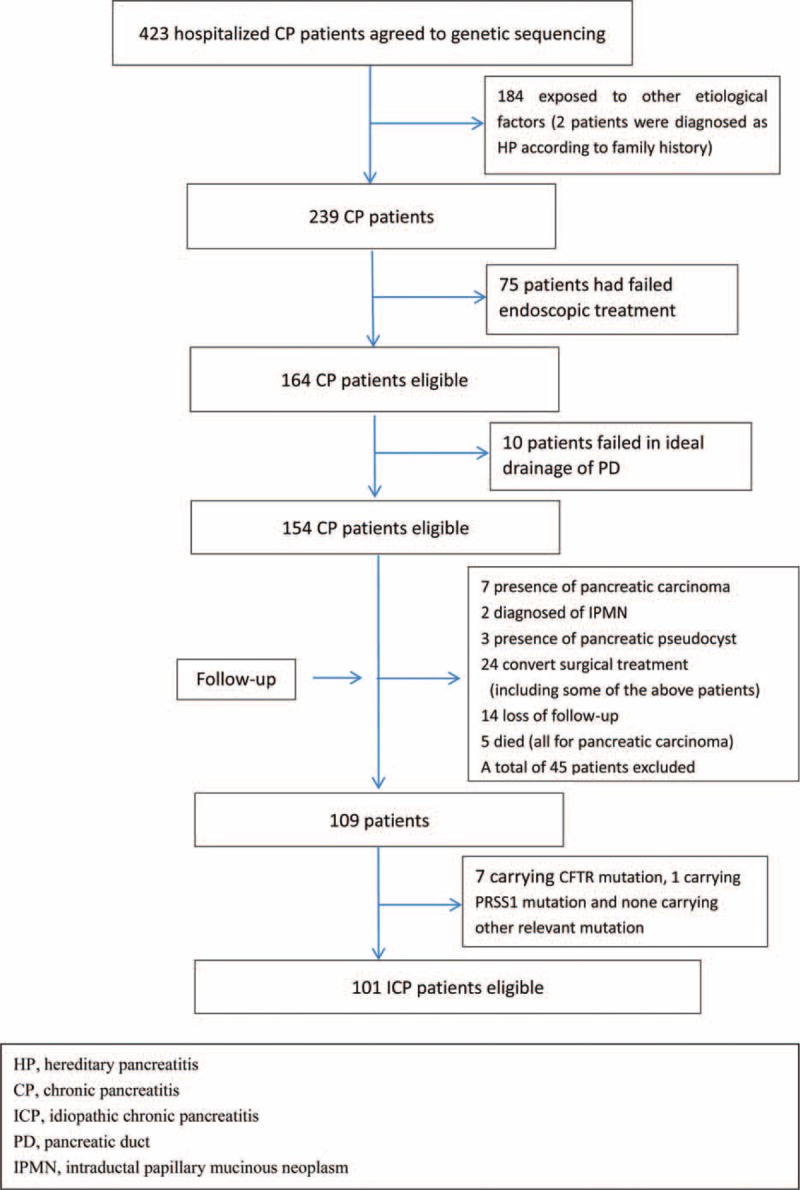
Study enrollment and long-term follow-up.

As Changhai endoscopic center is one of the leading endoscopic centers in China and the first institution equipped with extracorporeal shock wave lithotripsy facilities,^21^ some of these patients were referred to our hospital after a failed or otherwise insufficient previous endoscopic procedure. This, as well as a former sphincterotomy and/or stent insertion followed by unsuccessful endoscopic treatments, may lead to a biased outcome measurement. Thus, we only included patients without a history of pancreatic surgery or failed interventional procedures in the present study. As a result, a total of 164 suspected ICP patients were eligible for further analysis. Subsequently, during the follow-up from the day of enrollment to October 2013, 45 patients were excluded because of the development or confirmation of pancreatic cancer, the diagnosis of intraductal papillary mucinous neoplasm (IPMN) and the presence of pancreatic pseudocyst formation. Patients were also excluded if they received surgical operation or have failure of follow-up. A total of 109 ICP patients were eligible for genetic analysis (Fig. 1). Gene sequencing suggested that 1 patient carried PRSS1 N29I mutation and this patient was thus diagnosed of hereditary chronic pancreatitis (HP). In addition, 7 patients carrying cystic fibrosis transmembrane regulator (CFTR) mutation were excluded. Ultimately, 101 ICP patients without other relevant mutations were included in the final analysis.

At beginning of enrollment, baseline information was collected and 5 mL of peripheral blood was obtained from every patient, which was stored at −80 °C after anticoagulation treatment with EDTA. The duration of follow-up was calculated from the day of enrollment to the last contact before the cut-off date (October 2013). Patients were arranged visits at least every 6 months as outpatients or when their symptoms deteriorated and required a check-up. During each visit, patients underwent re-evaluation of clinical conditions, routine laboratory tests including fasting blood glucose and a 2 h glucose tolerance test, stool fat, and computed tomography (CT) and/or magnetic resonance cholangiopancreatography (MRCP) if necessary.^22^

This study was approved by the Ethics Committee of Changhai Hospital, Shanghai, and a written informed consent was obtained from each patient according to the ethical guidelines of the Declaration of Helsinki. Blood samples were obtained and processed under coded numbers to protect the privacy of patients.

### Diagnostic Criteria

The diagnosis criteria of CP are defined as follows: (a) typical clinical findings (recurrent epigastric pain or acute pancreatitis, etc); (b) indicative findings of imaging procedure (pancreatic calcifications, pancreatic stones, stenosis, or dilations of pancreatic stones, etc); (c) pathological findings (interstitial fibrosis, acinar loss, etc) if the patients have underwent endoscopic ultrasonography fine needle aspiration and H&E staining; (d) exocrine functional insufficiency; the presence of (b) or (c) alone can be diagnosed as CP; and the presence of (c) together with (d) needs further evaluation of the CP diagnosis.^23,24^ Idiopathic chronic pancreatitis was diagnosed after ruling out predisposing risk factors (such as alcohol abuse, trauma, previous medication, infection, metabolic disorders, and/or a positive family history).^10,23^ Hereditary chronic pancreatitis diagnosis is defined by the presence of a detected PRSS1 mutation (with or without clinical or radiological manifestations of chronic pancreatitis) or when the patient's family satisfies the requirements of the European Registry of Hereditary Pancreatitis and Pancreatic Cancer (EUROPAC).^25^

### Clinical Evaluation

At the time of enrollment, clinical data concerning a detailed clinical course, including age of disease onset, complications, and previous treatments, were recorded. Computed tomography and/or MRCP were used for the evaluation of pancreatic duct obstruction and/or stenosis, as well as pancreatic carcinoma and pseudocyst formation. Standardized evaluation of symptoms and laboratory investigations were performed. For patients who previously had successful endoscopic treatments at our center and had clear records of the procedure. We ask them to recall the history of treatments and symptoms. Types of clinical manifestations are defined as (1) no presence of pain; (2) episodes of mild to moderate pain, usually controlled by medication; (3) constant mild to moderate pain usually controlled by medication; (4) usually pain free with episodes of severe pain; (5) constant mild pain plus episodes of severe pain; (6) constant severe pain that does not change.^2,26^ In addition, we adapted the Izbicki pain scoring system to comprehensively evaluate the severity of pain and its impact on each individual.^27^ The Izbicki pain score was specifically designed for chronic pancreatitis and consists of subjective items such as intensity of pain and the frequency of pain attacks, as well as objective items including analgesic medication usage and duration of periods of inability to work. Patients were considered to have severe pain when the Izbicki pain score was >70.^27,28^

Pain relief at the end of follow-up was defined as complete (Izbicki pain score, ≤ 10), partial (Izbicki pain score, >10 after a decrease of >50%), and no relief. Endocrine function insufficiency was diagnosed when the fasting glucose level was >7.0 mmol per liter (126 mg per deciliter) and the glycated hemoglobin level was more than 6.5%.^28,29^ Exocrine function insufficiency was diagnosed if the stool fat was >7 g/24 h and needed pancreatic enzyme to control the symptom of steatorrhea.^30^ Changes in pancreatic function (both endocrine and exocrine) were evaluated by dividing the patients into 4 groups: (1) those who had pancreatic insufficiency at both baseline and follow-up and the glucose level or insulin requirement (pancreatic enzyme requirement) remains the same (insufficiency persisted); (2) those who did not have insufficiency at baseline but in whom insufficiency developed during follow-up as well as those who needed a lager dose of insulin (or pancreatic enzyme) or if the symptoms deteriorated with no change in treatments (insufficiency developed); (3) those who had insufficiency at baseline but not at follow-up as well as those who need smaller dose of insulin (or pancreatic enzyme) or if the symptoms relieved with no change in treatments (insufficiency resolved); (4) those who did not have insufficiency at baseline or follow-up (sufficiency persisted).^28^

### Endoscopic Treatments During Follow-Up

All endoscopic procedures took place at the Endoscopic Center of Changhai Hospital. Treatment plans were decided by attending physicians under the supervision of chief physicians in accordance with guidelines of treatments for chronic pancreatitis.^24^ Large pancreatic stones (>5 mm) or stones that are difficult to extract can be crushed via extracorporeal shock-wave lithotripsy (ESWL) with or without endoscopic retrograde cholangiopancreatography performed afterward.^7–9,31^ Pancreatic duct stent placement and dilatation was performed in cases of pancreatic duct stenosis. A stenosis was considered to be present if the pancreatogram showed a narrowing of the main pancreatic duct, dilatation of the duct by >5 mm proximal to the narrowing, and incomplete distal runoff of the contrast agent. Ideal drainage of the pancreatic duct was reached when complete runoff of contrast material was observed after removal of the stent and an extraction balloon could be passed through the pancreatic duct.^27^

The indications for selective operations or urgent operations are as follows: (a) indications for urgent operations: compilations of CP, including infections, bleeding, rupture of cysts, etc. (b) Indications for selective operations: (1) conservative or endoscopic treatments are useless; (2) pressure on nearby organs leading to biliary or duodenal obstructions intractable to endoscopic interventions or portal hypertension with hemorrhage; (3) pseudocysts, pancreatic fistula, or peritoneal effusions intractable to conservative or endoscopic treatments; (4) suspected malignancy.^24^

### Genetic Analysis

As previously described, mutational analysis of the SPINK1 gene was performed by direct sequencing.^19^ Genomic DNA was extracted from serum sample using the QIAGEN DNeasy Blood & Tissue Kit. We compared SPINK1 sequences to the GenBank reference sequences MIM 167790 in the National Center for Biotechnology Information database (http://www.ncbi.nlm.nih.gov).

The mutation scanning of CFTR, CTRC, and PRSS1 gene was performed using the direct sequencing methods as well as the HRM (high resolution melting) technique. Gene Scanning program in LightCycler^®^ 480 software version 1.5.0 (Roche Diagnostics, Germany) was used to perform melting curve analysis. PCR primers were designed in the intron regions and covered the whole parts of exon regions. We compared the CFTR PRSS1 and CTRC sequences to the GenBank reference sequences in the National Center for Biotechnology Information database (http://www.ncbi.nlm.nih.gov).^19^

### Statistical Analysis

An unpaired *t* test was used for quantitative comparisons. Additionally, the χ^2^ and Wilcoxon rank sum tests were used for qualitative data. Patients without endocrine (exocrine) insufficiency were excluded from the analysis at the end of follow-up. We included only patients with the presence of pain in the χ^2^ analysis, multivariate analysis, and the survival analysis. And factors influencing the efficacy of pain relief and pancreatic function failure were identified by cox regression. The criteria for variant entry are 0.05 and for variant removal is 0.1. Furthermore, we used the Kaplan–Meier method to individually evaluate the impact of the mutation on the prognosis of endoscopic therapy. A *P*-value of 0.05 was considered the cutoff for significance in *t* test and χ^2^ analysis. The SPSS (version 17.0) program was used for statistical analyses.

## RESULTS

### Baseline Information

A total of 101 patients completed genetic sequencing and follow-up between the time of enrollment and the cut-off date, and 58 (57.43%) patients had a SPINK1 c.194+2T>C mutation. The mean age at enrollment was 34 ± 16 years in patients with SPINK1 c.194+2T>C mutation and 42 ± 17 years in patients without the c.194+2T>C mutation. In a comparison between the 2 groups, patients with the SPINK1 c.194+2T>C mutation were enrolled at a significantly younger age (*P* = 0.015). No detectable difference was observed concerning the gender distribution of patients with or without the c.194+2T>C mutation.

Patients carrying a c.194+2T>C mutation had an onset of CP at a younger age (29 ± 15 vs 38 ± 17 years, *P* = 0.006). Comparison between the clinical status of patients with or without a SPINK1 c.194+2T>C mutation suggested a discrepancy in pain patterns (*P* = 0.019), and patients without SPINK1 c.194+2T>C mutation were more likely to have no presence of pain 12 (20.7%) compared with 1 (2.3%) patient without SPINK1 c.194+2T>C mutation. Additionally, the mean Izbicki pain score was higher in patients without the intron mutation (31 ± 21 vs 54 ± 21, *P* < 0.001). More patients suffered from exocrine function insufficiency in the c.194+2T>C mutation group (32.76% vs 13.95%, *P* = 0.030). Endocrine function insufficiency was found in 20 (34.48%) patients with c.194+2T>C mutation and 12 (27.91%) patients without the mutation, and no significant difference was identified (Table 1).

**TABLE 1:**
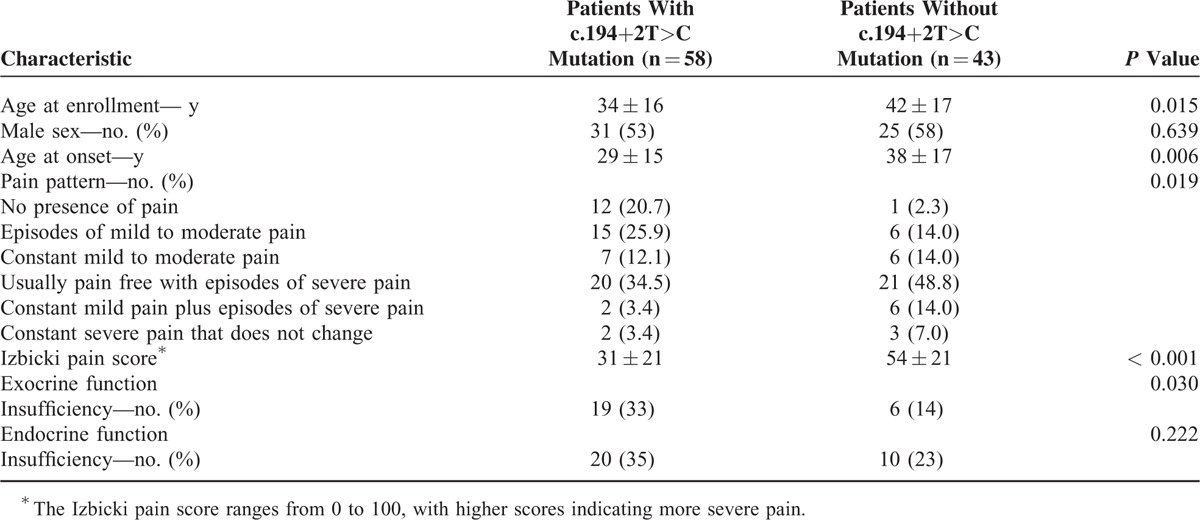
Demographic and Clinical Characteristics of Patients at Enrollment

### General Outcome at the End of Follow-Up

The median time of follow-up was 40 months (range, 18 to 62) and 39 months (range, 15 to 63) in patients with or without the c.194+2T>C mutation (*P* = 0.954). The Izbicki pain score appeared to be lower in patients without the intronic mutation, but no significant difference was found between groups. Among the 101 patients enrolled, 88 (87.13%) had the presence of pain and 49 (55.68%) among them had complete or partial pain relief at the end of follow-up. We excluded patients who has no presence of pain throughout the study and find that more patients without a SPINK1 c.194+2T>C mutation acquired pain relief at the end of follow-up, as compared to those without the mutated gene (*P* < 0.001).

A total of 28 ICP patients had exocrine function insufficiency before enrollment or during follow-up. After excluding patients without exocrine function insufficiency, comparison between patients with various mutation status revealed no statistically significant difference in function restore (*P* = 0.678). The diagnosis of pancreatic diabetes was confirmed in 39 patients before enrollment or during follow-up. Among them, endocrine function deterioration was found in more patients with the c.194+2T>C gene mutation than those without the mutation (0.001) (Table 2). More patients received insulin treatment (*P* = 0.003) and pancreatic enzyme supplement *P* = 0.011 in the SPINK1 intronic mutation group.

**TABLE 2:**
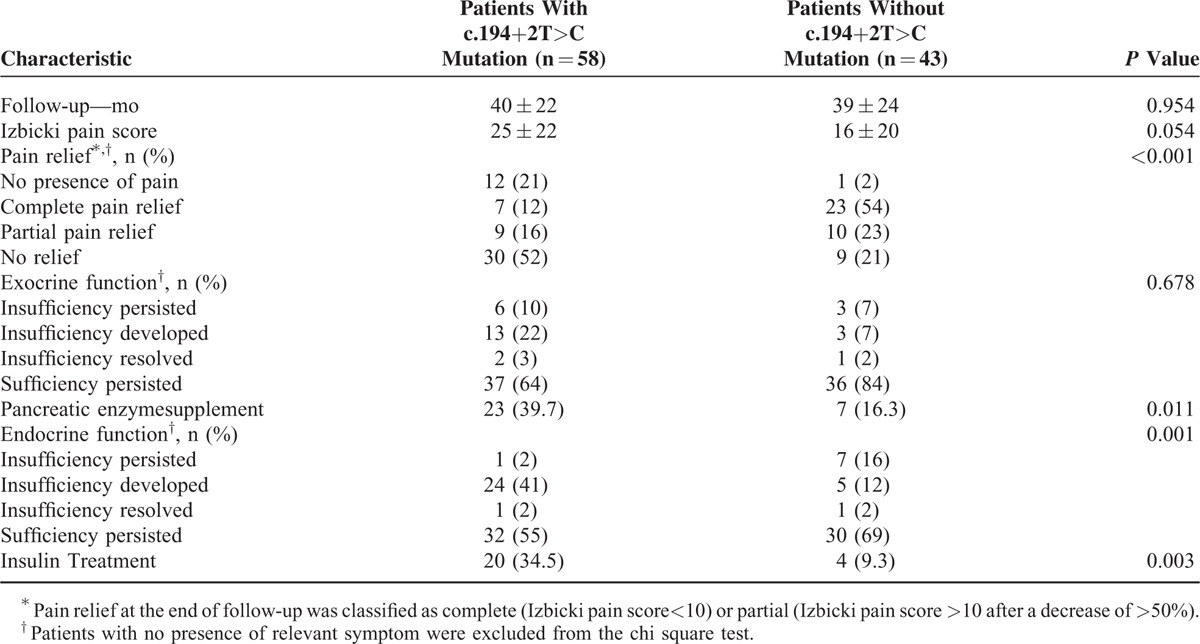
Outcomes at the End of Follow-Up

### Outcome of Endoscopic Treatments

Endoscopic treatments including ESWL and ERCP sphincterotomy, stone fragments or stone removal, nasopancreatic catheter placement, balloon dilatation, and stent insertion were performed strictly in accordance with guidelines for treatment of chronic pancreatitis.^24^ All patients included have shown marked changes on pancreatograms according to the Cambridge classification.^32^ A lithotripsy session immediately followed by endoscopic drainage was considered a single intervention. Successful endoscopic treatment was defined as ideal drainage of the pancreatic duct as described earlier. Among the 101 patients included, repeated endoscopic treatments were performed in 64 (63.37%) patients.

### Factors Associated With Pain Relief

**FIGURE 2:**
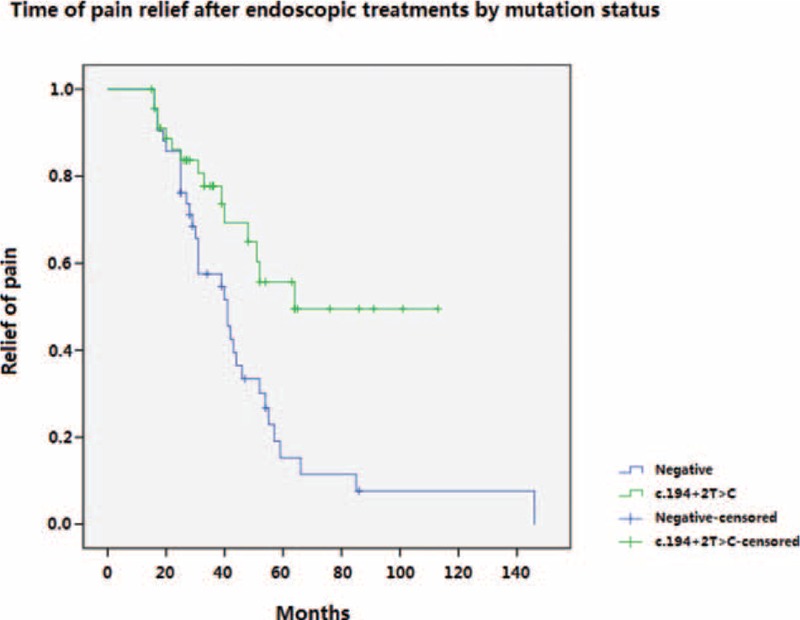
Time of pain relief after endoscopic treatments by mutation status. The patients without the SPINK1 c.194+2T>C mutation had an earlier relief of pain than those patients with the mutation (*P* = 0.003).

Multivariate analysis of patients with pancreatic pain suggested that the SPINK1 c.194+2T>C mutation (*P* = 0.011, HR = 0.434), severe pain before treatment (*P* = 0.005, HR = 3.095), and subsequent necessary endoscopic treatments (*P* < 0.001, HR = 0.224) are associated with pain relief after endoscopic treatments (Table 3). The Kaplan–Meier curve for pain relief over time in patients with or without a SPINK1 c.194+2T>C mutation suggested that patients without the intron mutation had an earlier relief of pain than those carrying the mutation (*P* = 0.003) (Fig. 2).

**TABLE 3:**
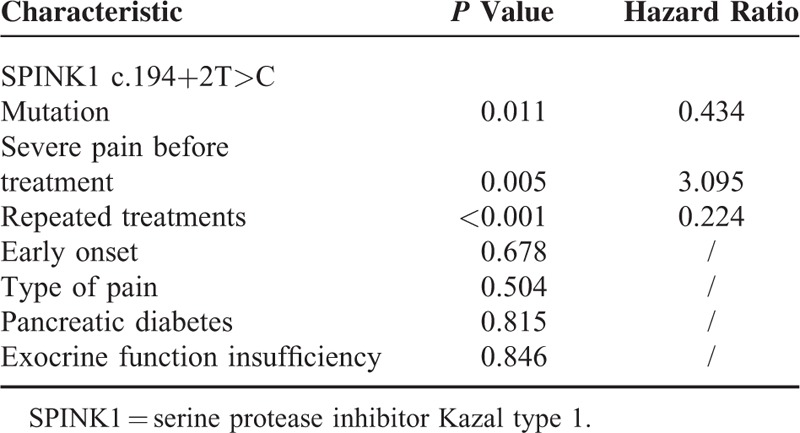
Multivariate Analysis Results of Factors Related to Pain Relief

## DISCUSSION

Endoscopic treatments including endoscopic retrograde cholangiopancreatography (ERCP) and Extracorporeal shockwave lithotripsy (ESWL) were considered the treatments after previous disappointing attempts at conservative treatment in controlling symptoms of chronic pancreatitis. To date, numerous studies from European, American, and Asian countries reported that 55% to 87% in all patients achieved pain relief.^4,5,7–9,31^ However, only 54.54% of all patients complained of complete pain re-acquisition or partial pain relief after a 3-year follow-up time, which is unexpectedly lower than previously reported data from other tertiary endoscopic centers. Stratified analysis revealed a possible explanation, with pain relief achieved in 34.78% of patients carrying SPINK1 c.194+2T>C mutation compared to 78.57% of patients without the mutation. Furthermore, multivariate analysis and survival analysis proved that a SPINK1 c.194+2T>C mutation could have led to a lower potential and a slower course of pain relief after endoscopic treatments. Thus, we inferred that the benefit after endoscopic treatments for patients without SPINK1 c.194+2T>C mutation may be a more rapid, effective, and sustained pain relief in comparison to those carrying the mutation. In addition, results of this study suggested that patients with Izbicki pain score >70 had a better chance of pain relief after ERCP and /or ESWL. It is known that patients with frequent and sever pancreatic pain episodes maybe a sign that the pancreas has reserved its exocrine function.^26^ To some extent, it can be interpreted that patients with frequent and sever pain may not be at the terminal stage of disease. By successfully decompression of the pancreatic duct the symptom may get ideal resolve. In the other hand, some patients experience pancreas “burn out” and experience little or even no pain.^26,33^ This may explain why patients with severe pain before treatment were more likely to acquire pain relief.

Theoretically, pancreatic duct drainage may restore exocrine function by a limited extent through obstruction remission and pancreatic fluid secretion improvement.^34^ Published data from American, European, and Asian groups have reported that 4% to 6% of patients acquired a re-establishment of exocrine function at 2 to 5 years after endoscopic treatments, and studies with longer follow-up periods suggested less of a benefit in pancreatic exocrine function.^5,7–9,28,31,34,35^ In our study, a very small number of patients gained exocrine function restoration after endoscopic treatment.

Up to 41% patients with SPINK1 c.194+2T>C mutation suffered a continuous deterioration of pancreatic endocrine function. A comparison between patients with varying SPINK1 c.194+2T>C mutation status suggested that the presence of the intronic mutation may lead to less benefit in pancreatic endocrine function through endoscopic treatments.

In the present study, intensity and frequency of pain were assessed by a valid scoring system that evaluates the overall damage of pain both objectively and subjectively in CP patients.^36^ In addition, we defined pain relief by strictly comparing pain scores at the time of enrollment and end of follow-up, eliminating the bias of imbalanced baseline data. The definition system was also applied in exocrine and endocrine function evaluation.^31^

Numerous genetic variations are considered the major pathogenic factors, with each variation conferring different degrees of risk. Studies from our group has convincingly demonstrated that a SPINK1 intron mutation (c.194+2T>C, OMIM ∗167790) was most commonly found among all currently seen mutations with a much higher incidence rate and no occurrence in healthy controls.^19,20^ By analyzing data from all ICP populations and in juvenile ICP patients, we found that the SPINK1 c.194+2T>C mutation may correlate with an earlier onset of diabetes, as well as a higher rate of presence of pancreatic stones. Kereszturi et al found that the c.194 + 2T > C intronic mutation abolished SPINK1 expression at the mRNA level and increased the risk of chronic pancreatitis by diminishing protective trypsin inhibitor levels.^37^ No study has yet elucidated the detailed pathway of the intron mutation altering and impacting the function of pancreatic stellate cells, acinar cells, and islet cells. Nevertheless, our study demonstrated a clear connection between c.194 + 2T > C mutation and clinical prognosis of endoscopic treatments. Further research should be devoted to the molecular mechanism by which this mutation influences pancreatic functional alteration and disease manifestation.

Because outpatients were unlikely to consent to genetic analysis and long-term follow-up, we enrolled only hospital in-patients who had underwent successful endoscopic treatment in our hospital. As a result, no eligible patients without a history of endoscopic treatment could confound the clarification of the exact roles that the intervention itself played in the course of ICP. We did not collect the data from patients receiving surgical treatments and patients who receive only conservative treatments as a comparison. This is a retrospective study, and the number of patients enrolled was limited by the diagnosis of idiopathic chronic pancreatitis, as well as the previous and unsuccessful endoscopic treatments.

## CONCLUSIONS

With the development of minimally invasive interventional treatments targeting chronic pancreatitis, a growing number of CP patients are hopeful of successful ESWL and ERCP to remove pancreatic stones, to alleviate pain, and to reverse pancreatic function failure. Clinical decisions primarily rely on radiology results. However, there has never been a predictive factor proven to be helpful in the evaluation of therapeutic effects. Judging from data in the present study, we conclude that the SPINK 1 c.194 + 2T > C mutation is a candidate pretreatment prediction indicator. Further studies should focus on the molecular mechanism of the mutation to confirm its influence on pancreatic cells and islet function. Additionally, prospective studies with larger sample sizes should be conducted to verify the clinical significance. Potentially, a commercialized detection kit could be used to aid in deciding treatment plans and predicting disease prognosis among patients.
